# Protein interaction, cytotoxic, transcriptomic and proteomic responses to structurally distinct EPAC1 activators in HUVECs

**DOI:** 10.1038/s41598-022-20607-8

**Published:** 2022-10-05

**Authors:** Jolanta Wiejak, Urszula Luchowska-Stańska, Pingyuan Wang, Jia Zhou, Pasquale Maffia, David Morgan, Graeme Barker, Stephen J. Yarwood

**Affiliations:** 1 Institute of Biological Chemistry, Biophysics and Bioengineering, Heriot-Watt University, Edinburgh, EH14 4AS UK; 2 Institute of Evolution and Marine Biodiversity, Ocean University of China, Qingdao, 266003 China; 3 Department of Pharmacology and Toxicology, University of Texas Medical Branch, Galveston, TX 77555 USA; 4 Institute of Infection, Immunity and Inflammation, University of Glasgow, Glasgow, G12 8TA UK; 5 Institute of Cardiovascular and Medical Sciences, University of Glasgow, Glasgow, G12 8TA UK; 6 Department of Pharmacy, University of Naples Federico II, 80131 Naples, Italy; 7 Institute of Chemical Sciences, Heriot-Watt University, Edinburgh, EH14 4AS UK

**Keywords:** Cell biology, Cell signalling, Proteomics, Transcription

## Abstract

The *N*-acylsulfonamide derivative, I942, represents the first non-cyclic nucleotide partial agonist of EPAC1. This was soon followed by the identification of the I942 analogues, PW0381, PW0521 and PWO577 and a series of benzofuran oxoacetic acid EPAC1 activators, SY006, SY007 and SY009. Protein interaction, cytotoxicity and EPAC1 activation assays applied here identify PWO577 and SY007 as being effective EPAC1 binders that are well tolerated in HUVECs at concentrations greater than 100 μM and up to 48 h incubation and are effective activators of transfected EPAC1 in U2OS cells. Using RNAseq in HUVECs we show that PWO577 and SY007 regulate approximately 11,000 shared genes, with only few differential gene changes being “off-target”. The genes significantly regulated by both PWO577 and SY007 included a subset of genes normally associated with endothelial activation, including ICAM1, MMP1 and CCL2. Of these, only the expression of MMP1 was markedly increased at the protein level, as determined by LC–MS-based proteomics. Both PWO577 and SY007 suppressed IL-6-induced STAT3 activation and associated downstream gene expression, including inhibition of SOCS3, STAT3, IL6ST and JAK3 genes. Together these results demonstrate the utility of structurally distinct, specific and non-toxic EPAC1 activators. Future modifications will be aimed at eliminating the few noted off-target effects.

## Introduction

The second messenger, cyclic AMP, initiates a multitude of downstream signalling processes in cells through the activation of the sensor proteins, protein kinase A (PKA)^1^, cyclic nucleotide-gated ion channels^2,3^, exchange proteins activated by cyclic AMP (EPAC1 and EPAC2)^4,5^, Popeye domain-containing proteins (POPDC)^6,7^, or the cyclic nucleotide receptor involved in sperm function (CRIS)^8^. Among these, PKA and EPAC are implicated as being the prime mediators of cyclic AMP effects in cells^9^. Therefore, to fully elucidate the mechanisms underlying the various actions of cyclic AMP, differentiation between PKA- and EPAC-mediated signalling events is essential. To facilitate this, selective research tools, in the form of cyclic nucleotide agonists, have been developed, including 6-Bnz-cAMP^10^, which preferentially activates PKA, as well as the EPAC-selective cyclic AMP analogues, D-007^11^ and S-220^12^, which selectively activate EPAC1 and EPAC2, respectively^12^. Unfortunately, the practical use of S-220 in vivo is limited, since activation of EPAC2, but not EPAC1, causes arrhythmia and reduced cardiac function in animal models^13,14^. Additionally, Hothi et al. reported that activation of EPAC1 in cardiomyocytes with D-007 is associated with disturbed calcium homeostasis and arrhythmia^15^, whereas genetic or pharmacological inhibition of EPAC1 prevented arrhythmia in mice^16,17^. Moreover, plausible analogue syntheses from these compounds are limited to sugar protections and substitution in the adenosine eight-position^18^.

To address this, we have applied high throughput screening approaches (HTS) to identify novel, small molecule, non-cyclic nucleotide (NCN) EPAC1-selective activators^19–21^, including I942, which displayed molecular parallels with the well-established sulfonylurea drug class making it amenable to tractable development as a bioavailable ligand^20^. Moreover, I942 effectively stimulates EPAC1, but not EPAC2, activity towards Rap1, with no effect on PKA activity^20^, leading to up-regulation of SOCS3 and suppression of IL-6-induced STAT3 activation and VCAM1 expression in human umbilical vein endothelial cells (HUVECs)^22^. Subsequently we developed an expanded, ca. 50 compounds, library of I942 analogues^21^, the most effective of which (PW0381, PW0521 and PWO577) display sub-µM binding potencies towards EPAC1 and stimulate EPAC1 activity in cells^21^. In addition, we applied ultra-HTS, which led to the further identification of a novel, chemically distinct benzofuran oxaloacetate series of EPAC1-selective activators^19^, with similar levels of efficacy towards Rap1 (SY006, SY007 and SY009). Under physiological conditions, cyclic AMP activates EPACs through disruption of an “ionic lock” moiety which stabilises the inactive conformation by the cAMP phosphate. Both series of compounds feature an anionic phosphate-mimetic, an oxalate in the case of the SY series, and an *N*-acylsulfonamide (N–H pKa ~ 5) for the PWO series, which are proposed to destabilise the EPAC1 inactive conformation via the same mechanism^23^.

Here we carried out EPAC1 binding and denaturation assays together with in vitro cytotoxicity testing of these identified lead ligands, from the *N*-acylsulfonamide and benzofuran oxoacetic acid series, to eliminate toxic compounds at an early stage^24,25^. HUVECs were chosen as a model for in vitro cytotoxicity testing to provide a more authentic representation of actual target tissues and might therefore allow for the detection of cytotoxic effects at lower compound concentrations. PWO577 and SY007 were selected as the best performers in EPAC1 binding and cytotoxicity assays and were further compared for their abilities to regulate global gene expression by RNAseq and LC–MS proteomics as well as for their ability to inhibit IL-6 activated STAT3 and downstream gene expression. Results demonstrate that PWO577 and SY007 exert qualitatively similar gene responses in HUVECs despite having chemically distinct core structures.

## Results

### Effects of EPAC1 activators on protein stability

**Figure 1:**
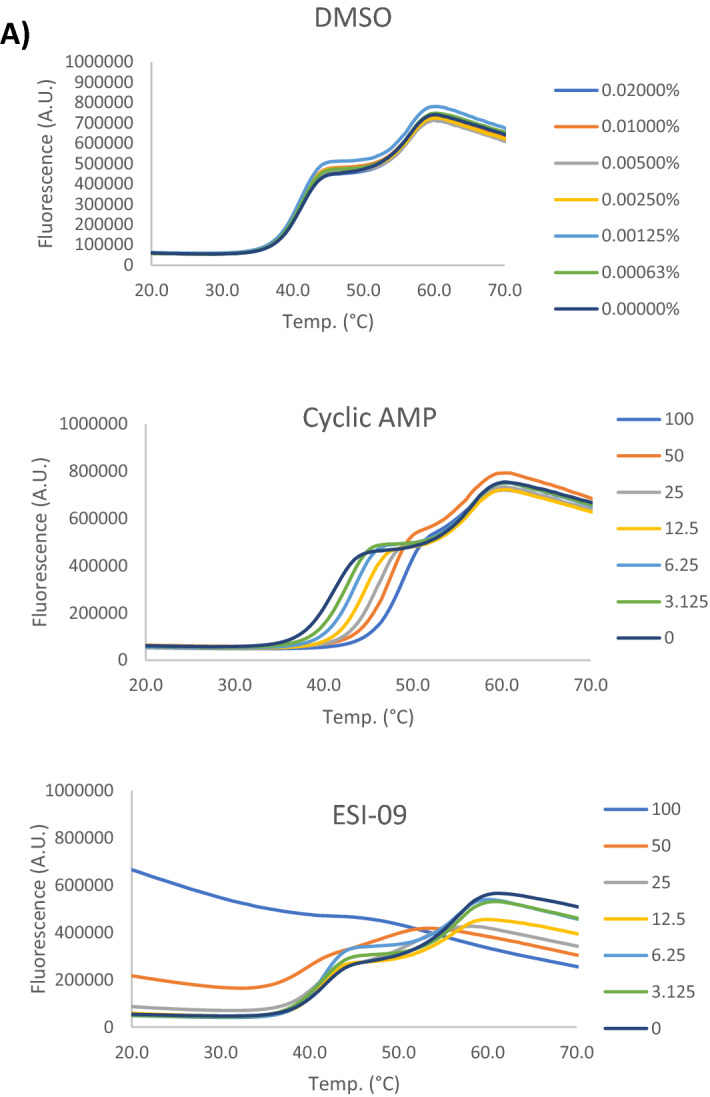

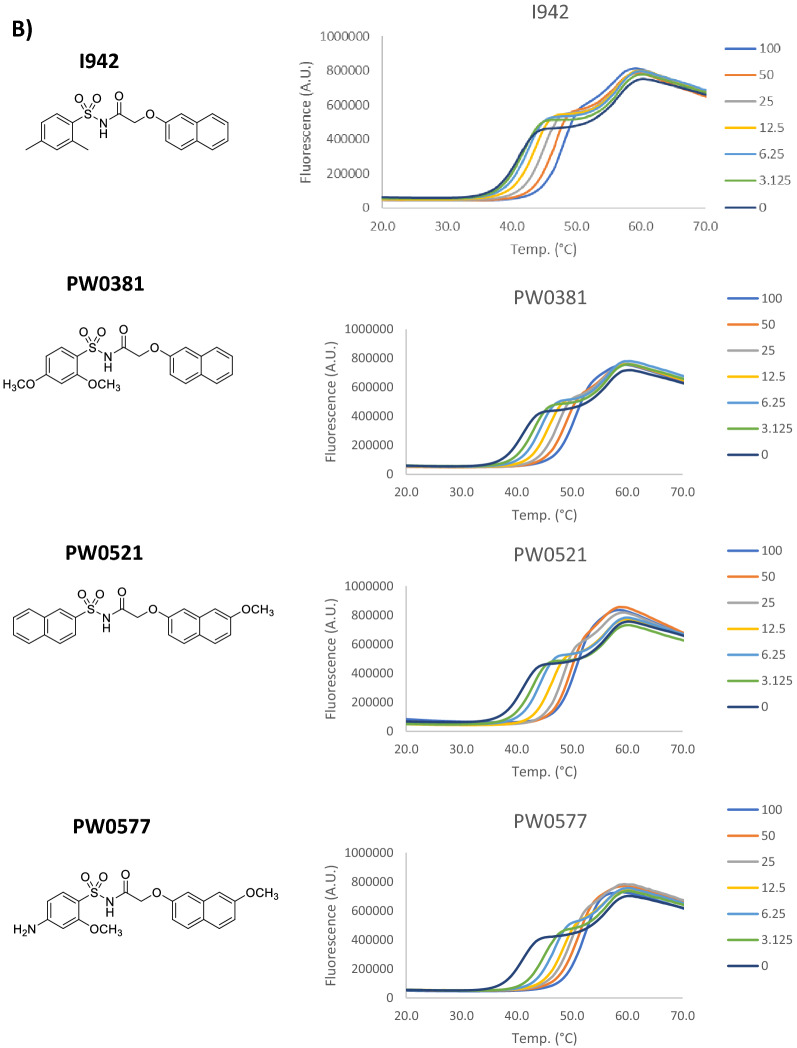

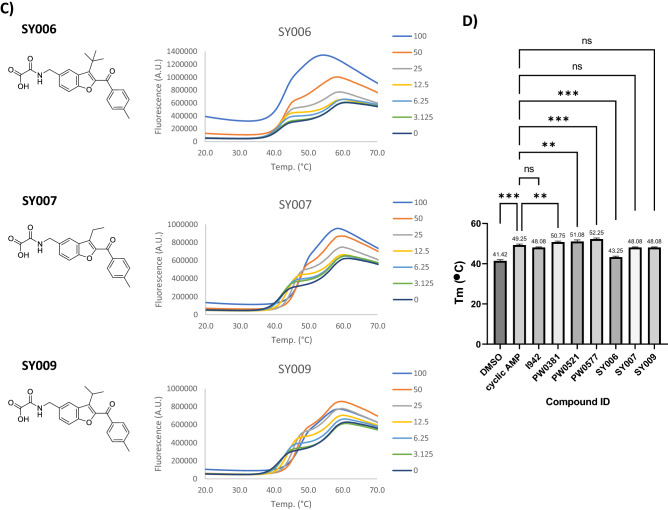
Thermal shift profiles of EPAC1 ligands. (**A**) A dilution series of cAMP or ESI-09 (3.125–100 µM range) plus diluent (DMSO; concentrations as indicated in graph) were prepared and combined with 2 µM GST-EPAC1-CNBD and 10 × SYPRO Orange dye, in a PCR-compatible microplate. Plates were incubated overnight at 4 °C, and then subjected to a standard temperature gradient programme using a real-time PCR machine, with a temperature range of 11–80 °C, ramping up in 0.5 °C increments, with a 30-s hold at each temperature Changes in fluorescence of SYPRO Orange then were monitored. A 20–70 °C range is shown in the graphs. Presented data is an average of three independent experiments. (**B**) Thermal shift assays (TSA) were carried out on a dilution series of I942 (3.125–100 µM range) and selected chemical analogues, as described in the legend to (**A**). (**C**) TSAs were carried out on a dilution series of “SY” series analogues (3.125–100 µM range) and selected chemical analogues, as described in the legend to (**A**). (**D**) Tm values for 100 μM ligand concentration from the TSAs carried out in (**A**–**C**) are displayed as a bar graph. Significant differences in Tm values relative to cyclic AMP are indicated, **p < 0.01 and ***p < 0.001, or ns, for no significant difference (n = 3–5).

Nonspecific interactions and associated protein-destabilising properties of small molecule ligands can lead to false-positive hits in screening studies. To address this concern for our previously identified EPAC1 activators, we tested I942^20^ and the identified I942 analogues, PWO381, PWO521 and PWO577^21^ (Fig. 1B), plus the SY series compounds SY006, SY007 and SY009^19^ (Fig. 1C), for their ability to interact with and effect protein stability of the EPAC1 cyclic nucleotide-binding domain (CNBD), using thermal shift assays (TSA)^26^. TSA monitors protein thermal denaturation in the presence of a potential ligand using an environmentally sensitive fluorescent dye, such as SYPRO Orange. Increasing temperature leads to protein unfolding and exposure of hydrophobic regions that SYPRO Orange can bind to, promoting an increase in fluorescence signal^26^. The resulting melt curves provide information on ligand binding, which usually stabilises the protein and shifts the melting temperature (Tm) towards higher values, but also on potential destabilising properties of the compound. At low temperatures, proteins should be natively folded, which is accompanied by a minimal SYPRO Orange signal. If high fluorescence values are observed instead, it means that the test compound might be denaturing.

Recombinant GST-EPAC1-CNBD protein, test ligand dilution series, and SYPRO Orange dye, were combined in a PCR-compatible microplate. Cyclic AMP was also used in addition to the test EPAC ligands, as a protein-binding and protein-stabilising control, while the EPAC1 antagonist, ESI-09, was employed as a protein-denaturing control as previously described^27^ (Fig. 1A). Vehicle controls (DMSO) were included as well to account for potential effects of the ligand solvent (Fig. 1A). As shown in Fig. 1A, cyclic AMP causes a dose-dependent shift in GST-EPAC1-CNBD Tm towards higher values, which means it increases the thermal stability of the protein by binding to it specifically, as expected. At the same time, high concentrations of ESI-09 (100 µM, and to a lesser extent, 50 µM) were found to distort the shape of the melt curve and induce protein unfolding at low temperatures, which is manifested by high fluorescence signal in this region, confirming the denaturing properties of this EPAC inhibitor at high concentrations, as previously described^27,28^. Thermal profiles for I942, PWO381, PWO521 and PWO577 (Fig. 1B) and SY007 (Fig. 1C) retain correct shapes and low fluorescence at low temperatures, demonstrating that none of the compounds negatively affects the stability of GST-EPAC1-CNBD. All test compounds induce a dose-dependent increase in the Tm value, resembling the stabilising effect of the endogenous ligand, cyclic AMP. All Tm values at 100 µM ligand concentration were compared using one-way ANOVA with Tukey’s post hoc test to detect statistically significant differences (Fig. 1D). Consistent with previous 8-NBD-cAMP binding assay data^21^, PWO381, PWO521 and PWO577 are characterised by improved binding affinity, as 100 µM concentrations of these compounds induce a significant increase in ∆Tm of 9 °C, 9.1 °C and 10.5 °C, respectively, compared to cyclic AMP (Fig. 1D). SY007 and SY009 produced a comparable increase in Tm to cyclic AMP at 100 µM, but SY006 was observed to exert denaturing properties on GST-EPAC1-CNBD at this concentration (Fig. 1C).

### Effects of EPAC1 activators on cell cytotoxicity

Having determined the impact of EPAC1 activators on the stability of the EPAC1 CNBD, we next explored whether the compounds displayed unwanted cytotoxic effects in HUVECs, which we routinely use for inflammatory assays^22^. To do this, we employed a multiplex method utilizing three fluorescent viability probes, Alamar Blue, CFDA-AM and Neutral Red as described in Material and Methods. Before investigating the cytotoxicity profiles of I942 and lead compounds, their autofluorescence at all three assay wavelengths was tested to rule out the possibility of any of them interfering with the readings (results not shown).

**Figure 2:**
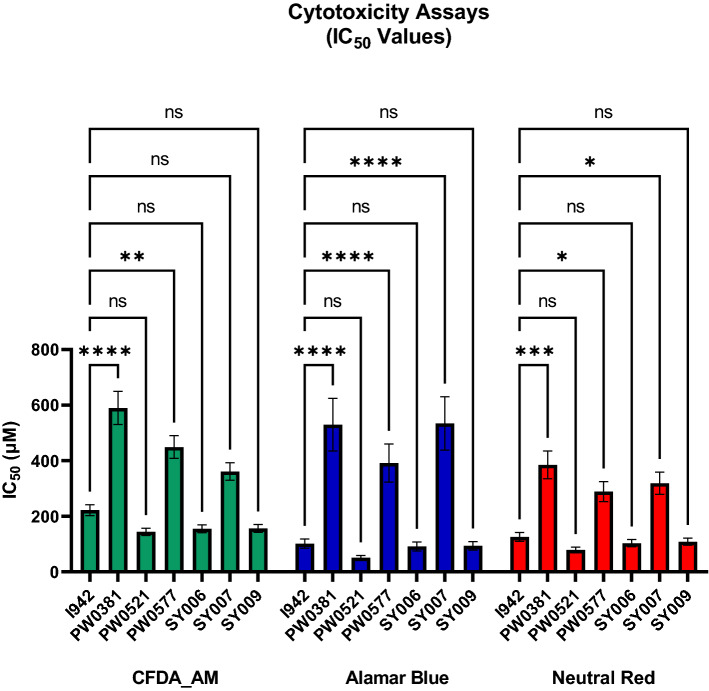
Cytotoxic effects of EPAC1 Activators in HUVECs. HUVECs were treated with a concentration range of I942 (3.9–500 µM) for 24 h, followed by incubation with a mixture of Alamar Blue and CFDA-AM dyes for 30 min in the dark. Fluorescence was then measured at 532/590 nm (for Alamar Blue) and at 485/535 nm (for CFDA-AM) ex/em wavelengths. Subsequently, cells were incubated with Neutral Red dye for 1 h in the dark, followed by cell solubilization and fluorescence reading at 532/645 nm ex/em wavelengths. Curves were fitted to the data collected in all three assays to obtain EC50 values, which are plotted here as a bar graph. Data are presented as means ± SEM (n = 3).

HUVECs were exposed to I942, PWO381, PWO521, PWO577, SY006, SY007 and SY009 with a concentration range of 3.9–500 µM, for 24 h and then IC_50_ values were calculated and compared between the compounds, for Alamar Blue, CFDA-AM and Neutral Red assays, respectively. Statistical differences between these values were determined using one-way ANOVA with Tukey’s post hoc test (Fig. 2). The Alamar Blue assay demonstrated that I942, PWO521, SY006 and SY009 have relatively low IC_50_ values and are therefore significantly less tolerated than either PWO381, PWO577 or SY007. The results from the Neutral Red and CFDA-AM cytotoxicity assay (Fig. 2) again follow the same trend as the Alamar Blue assay and show that exposure to PWO381, PWO577 and SY007 is better tolerated in HUVECs than treatment with the other test ligands (Fig. 2). Overall, the presented results confirm lower cytotoxicity of PWO381, PWO577 and SY007, relative to I942, PWO521, SY006 and SY009. SY007 was therefore carried forward for further assays due to its tight interactions with EPAC1, lack of protein denaturing and tolerance in cytotoxicity assays. PWO577 was also carried forward as a representative from the PWO series due to its improved ability to activate EPAC1 in vitro, over PWO381, as demonstrated previously^21^.

### Transcriptomic and proteomic responses to EPAC1 activators

**Figure 3:**
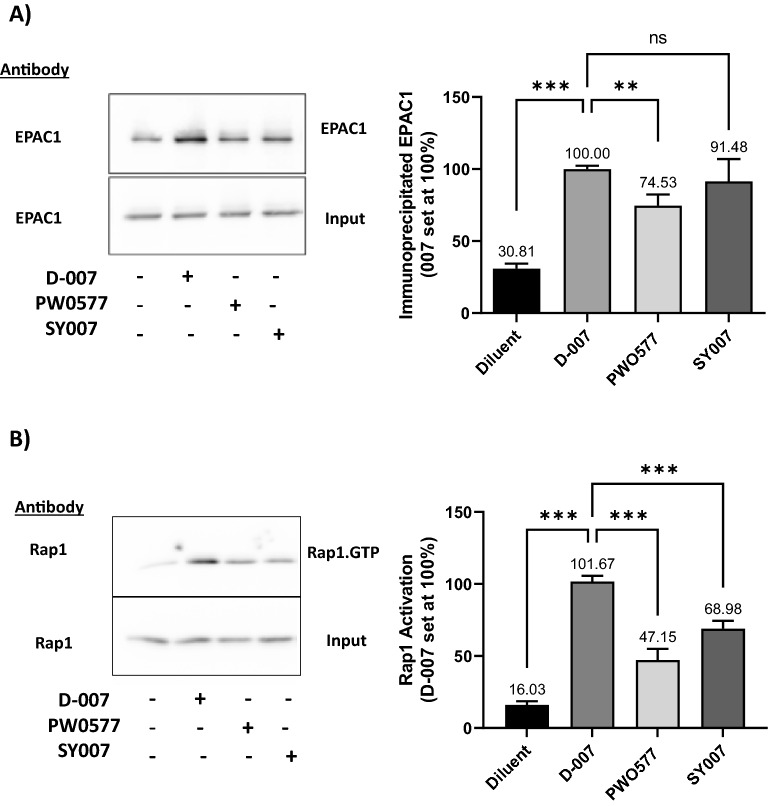
Effects of EPAC1 Activators on EPAC1 and Rap1 Activation. (**A**) U2OS cells transfected with EPAC1 were preincubated for 10 min with 100 μM of EPAC1 activators, PWO577, SY007 or D-007. Active EPAC1 was then isolated from cells by immunoprecipitation with the activation selective EPAC1 (DE3) antibody and detected by immunoblotting with the same antibody, as described in “Materials and methods”. Significant increases in active EPAC1 levels relative to D-007-treated cells are indicated; **p < 0.01 and ***p < 0.001 or ns, for no significant difference (n = 5–7). (**B**) U2OS cells transfected with EPAC1 were preincubated for 10 min with 100 μM of EPAC1 activators, PWO577, SY007 or D-007. Active Rap1 was then isolated from cell lysates, immunoblotted and compared to total Rap1 levels, as described in “Materials and methods”. Data are presented as means ± SEM (n = 5–7). Significant increases in active Rap1 levels in comparison to D-007-treated cells treated cells are indicated, p < 0.001 (n = 5–7).

**Figure 4:**
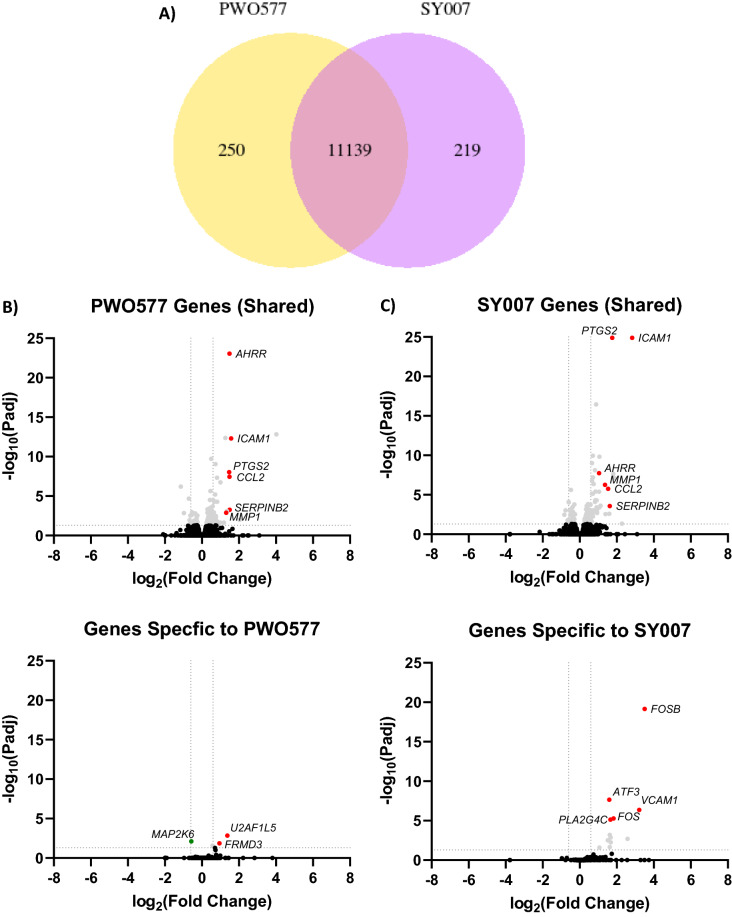

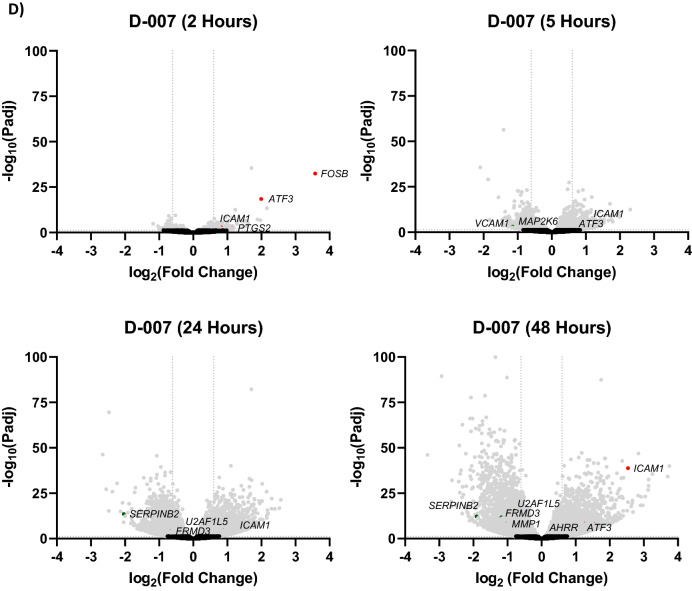
“On”- and “Off”-target Gene Regulation by PWO577 and SY007 in HUVECs. To identify gene regulation by EPAC1 activators, confluent HUVECs were stimulated for 16 h with PWO577 and SY007. Total RNA was then extracted and processed for RNAseq and plotted as a Venn diagram (**A**) or volcano plots (**B**,**C**), as described in “Materials and methods”. In the upper panel of (**B**,**C**) the data represents gene expression regulated by both PWO577 and SY007, encompassing 11,139 shared gene expression changes, whereas the lower panel demonstrates genes regulated solely by either PWO577 (**B**) or SY007 (**C**), representing either 250 or 219 gene expression changes, respectively. Significant gene expression changes (p > 0.05) are shown in light grey with changes in gene expression greater than 2-fold indicated either red (increase in gene expression) or green (decrease in gene expression). Non-significant gene changes are presented in black. In (**D**), previously published data^29^ has been re-analysed to demonstrate changes in gene expression promoted by a time-course of D-007 treatment. Named genes represent those that were also regulated by either PWO577 or SY007.

PWO577 and SY007 were next tested for their ability to activate EPAC1 in U2OS cells expressing EPAC1 using immunoprecipitation with an activation-selective antibody and Rap1 activation assays (Fig. 3). This demonstrated that although both compounds fully engaged comparably with EPAC1, as demonstrated by immunoprecipitation (Fig. 3A), the levels of stimulated Rap1 activation induced by PWO577 and SY007 (Fig. 3B) were not significantly comparable to those achieved with D-007, indicating partial agonist effects of PWO577 and SY007 towards Rap1 (Fig. 3). Given that PWO577 and SY007 exert similar levels of EPAC1 activation in cells, we next applied RNAseq to examine whether the transcriptomic response to PWO577 and SY007 was also similar (Fig. 4; Supplementary Data). For this, HUVECs were stimulated for 16 h in the presence or absence of EPAC1 activators then total RNA was extracted from cells; this was used for RNA library preparation and subsequently sequenced using the Illumina platform, as described in “Materials and methods”. The gene expression level is estimated by the abundance of transcripts (count of sequencing) that mapped to genome or exon locations. Comparison of the abundance of transcripts from each treatment grouped demonstrated that the majority of 11,139 transcripts identified were regulated by both PWO577 and SY007 (Fig. 4A; Supplementary Data), indicating that both EPAC1 activators exert similar transcriptomic responses in HUVECs. However, 250 transcripts appeared to be regulated specifically by PWO577 and a further 219 by SY007. It could be concluded that these changes represent “off-target” effects, but it cannot be ruled out that the 2 ligands may activate EPAC1 in different manners.

**Figure 5:**
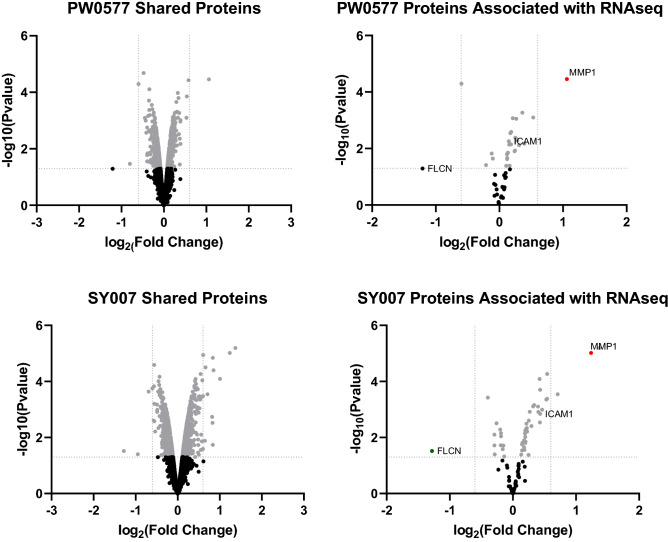
Proteomic Analysis of HUVECs stimulated with either PWO577 or SY007. HUVECs were stimulated for 16 h with either PWO577 or SY007 and then cell pellets were prepared. Protein extraction was then carried out on cell pellets followed by labelling, tryptic digest and analysis by LC–MS as described in “Materials and methods”. 5555 changes in protein expression, following PWO577 (upper left) or SY007 (upper right) treatment, are shown as volcano plots in the left-hand panels. Significant changes in protein expression (p > 0.05) are shown in light grey. Protein expression changes, associated with genes identified in Fig. 4 as being regulated by either PWO577 or SY007, are plotted in the right-hand panels. Significant protein expression changes (p > 0.05) are shown in light grey with changes in protein expression greater than 2-fold indicated either red (increase in gene expression) or green (decrease in gene expression). Non-significant protein changes are shown in black.

After gene expression quantification, statistical analysis of the expression data was carried out to screen for genes whose expression levels are significantly different in the different treatment conditions (Fig. 4B,C; Supplementary Data). Volcano plots indicated 100 significant gene changes from PWO577 treated cells (Fig. 4B) and 142 from SY007 treated cells (Fig. 4C). Only a few “off target” significant gene changes were identified for PWO577 and SY007 however proteomic analysis indicated that these “off target” genes were not translated into significant changes in protein expression in these cells (Fig. 5; Supplementary Data). Moreover, many of the “on-target” gene changes induced by treatment by both PWO577 and SY007, were not translated into changes in protein expression (e.g., CCL2; Fig. 5) and the majority of gene changes that were translated into changes in protein expression, were small and didn’t breach the twofold barrier for acceptance (e.g., ICAM1; Fig. 5). In fact, only MMP1 was regulated by both PWO577 and SY007 to produce a significant upregulation in protein expression that was greater than twofold (Fig. 5). These results suggest that while PWO577 and SY007 exert significant effects on gene activity in HUVECs, the effects on translation of target genes are modest. It should also be noted that re-analysis of previously published RNAseq data^29^ from D-007 stimulated HUVECs (Fig. 4D; Supplementary Data), demonstrated that ICAM1 was also significantly upregulated following D-007 treatment at all time points tested (Fig. 4D). Whereas some of the gene changes that were upregulated in response to PWO577 and SY007 (e.g., MMP1 and SERPINB2), were downregulated in response to D-007 treatment for 48 h (Fig. 4D). Moreover, certain “off-target” actions, including upregulation of FOSB and ATF3, which was associated with SY007 treatment, were also upregulated by 2 h D-007 treatment (Fig. 4D). The reason for the similarities and differences between D-007 treatment, and treatment with either PWO577 or SY007, is not yet known but may be due to differences in cell responses to either a full agonist (D-007) or partial agonists (PWO577 and SY007) and the resulting consequences for the dynamics of EPAC1 activation in HUVECs.

### Inhibition of IL-6 signalling by EPAC1 activators

We have previously shown that I942 effectively stimulates EPAC1 to promote up-regulation of the SOCS3 gene and suppression of IL-6-induced STAT3 activation and VCAM1 expression in HUVECs^22^. It is IL-6 receptor “trans-signalling”^30^ that is thought to underlie the pro-inflammatory actions of IL-6 in a variety of diseases, including atherosclerosis^31^. During *trans*-signalling, IL-6 binds to soluble forms of IL-6R (sIL-6R) allowing activation of gp130, leading to receptor clustering and activation of the JAK-STAT3 and ERK, MAPK and PI3K signalling pathways. Of these, it is activated STAT3 that then homodimerises and translocates to the nucleus, where it acts as a transcription factor for the induction of pro-inflammatory IL-6-responsive genes^32–34^.

**Figure 6:**
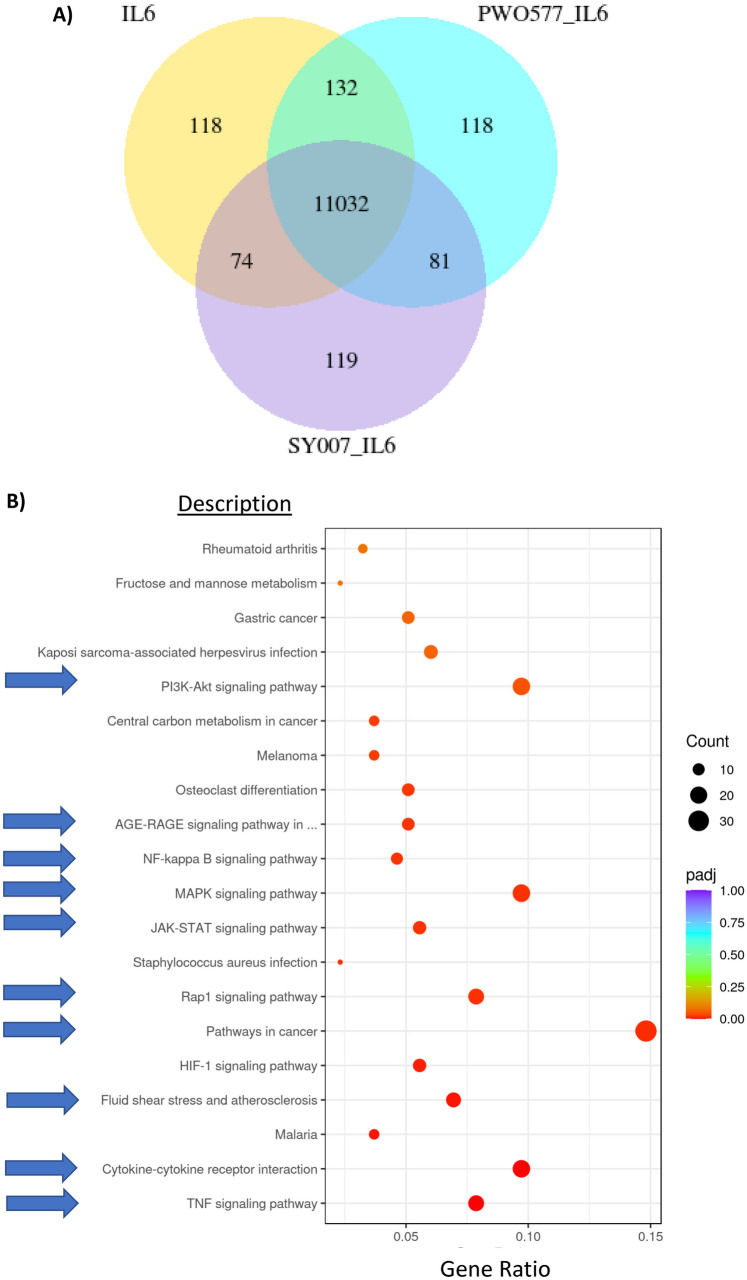
Analysis of gene expression changes induced by IL-6 in the presence of PWO577 or SY007 in HUVECs. (**A**) To identify regulation of IL-6 gene expression by EPAC1 activators, confluent HUVECs were stimulated for the indicated times with IL-6 (5 ng/ml) plus sIL-6Rα (25 ng/ml) in the presence or absence of the indicated concentrations or D-007, PWO577 or SY007. Total RNA was then extracted and processed for RNAseq and plotted as a Venn diagram. (**B**) Gene expression changes from IL-6/sIL-6Rα-stimulated cells, compared to diluent treated control cells, were further analysed to identify common biological functions associated with gene expression changes by comparison with the Kyoto Encyclopaedia of Genes and Genomes (KEGG) database^51,52^. The occurrence of individual gene ontologies (Gene ratio) and their significance (p-adjust) are displayed as a dot plot. Arrows indicate the gene expression changes associated with functions associated with cytokine action, cancer and inflammatory pathways.

**Figure 7:**
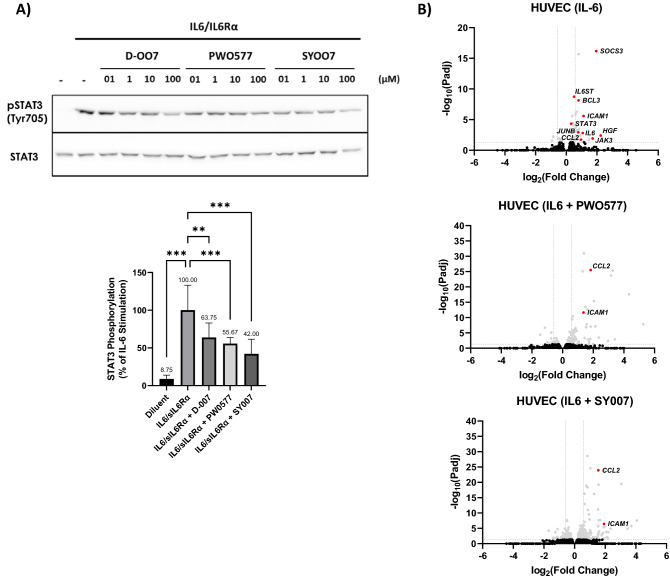
Impact of PWO577 and SY007 on IL-6 Signalling in HUVECs. (**A**) HUVECs were stimulated for the indicated times with IL-6 (5 ng/ml) plus sIL-6Rα (25 ng/ml) in the presence or absence of the indicated concentrations or D-007, PWO577 or SY007. Cell extracts were then immunoblotted with antibodies to pSTAT3 (Tyr705) or total STAT3. Densitometric values from 3 separate immunoblots, at 100 μM ligand concertation, are shown in the bar graph in the lower panel with significant changes STAT3 phosphorylation, relative to cells stimulated with IL-6/sIL-6Rα alone, being indicated: **p < 0.01 and ***p < 0.01 (n = 3). (**B**) HUVECs were stimulated with IL-6/sIL-6Rα, as described above, in the presence or absence of either PWO577 or SY007, as indicated. RNAseq was then performed, as described in “Materials and methods”, and the resulting gene expression changes shown as volcano plots. Significant gene changes (p < 0.05) are shown in light grey whereas non-significant gene changes are shown in black. Significant changes in gene induction associated with IL-6 signalling are shown in red.

Here we use RNAseq and KEGG pathway analysis to show that in HUVECs, IL-6 stimulation leads to the regulation of more than 11,000 genes (Fig. 6; Supplementary Data), many of which are involved in cytokine action, cancer and inflammatory pathways, including Rap1, PI3K, MAPK and JAK-STAT signalling (indicated by arrows in Fig. 6B). Moreover, many of the genes regulated by IL-6 are also regulated by PWO577 and SY007 (Fig. 6B). In terms of gene regulatory pathways, we show that 100 µM D-007, PWO577 or SY007 significantly inhibited IL-6-stimulated STAT3 activation (Fig. 7A). Moreover, PWO577 and SY007 also inhibited downstream IL-6 gene targets in HUVECs, including SOCS3, IL6ST, STAT3 and JAK3 (Fig. 7B; Supplementary Data). Interestingly, neither PWO577 or SY007 were able to down-regulate IL-6-induced ICAM1 and CCL2 gene expression, consistent with the ability of the two EPAC1 activators to directly upregulate the expression of these genes (Fig. 5B), although it is likely that this does not lead to a sizeable upregulation of the protein products of these genes as shown by proteomics in Fig. 5.

## Discussion

To address concerns about false-positive hits from screens, resulting from non-specific protein-denaturing properties of compounds on EPAC1^27^, we tested the effects of I942 and the three identified lead analogues, PWO381, PWO521 and PWO577, on EPAC1 stability in vitro. TSA experiments were carried out and thermal denaturation profiles of recombinant GST-EPAC1-CNBD in the presence of increasing compound concentrations were analysed (Fig. 1). Importantly, no distortions of the protein melt curves, which are characteristic for protein-denaturing agents, such high concentrations of ESI-09^27^, were observed at low temperatures in the presence of test compounds. It can therefore be concluded that I942 and selected analogues do not negatively affect the natively folded state of recombinant GST-EPAC1-CNBD and that protein-destabilising properties of these compounds can be ruled out with high probability. Moreover, I942, PWO381, PWO521 and PWO577 induced a dose-dependent protein stabilisation, a typical indicator of ligand binding, which manifests itself in a shift of Tm towards higher values. This confirmed the protein–ligand interactions and relative binding affinities observed previously using 8-NBD-cAMP competition assays^19–21^ and assured us that none of the three newly identified EPAC1 ligands was a false-positive hit.

For a reliable and comprehensive cytotoxicity assessment, it is strongly recommended that more than one type of assay is used^25^. The method we selected meets this requirement, as it comprises Alamar Blue, CFDA-AM and Neutral Red assays, which test for different viability and cytotoxicity indicators, including effects on metabolic activity, membrane integrity or lysosomal function. In general, from the *N*-acylsulfonamide series, compounds PWO381 and PWO577 were significantly better tolerated by HUVECs than I942 and PWO521. In the case of benzofuran oxoacetic acid ligands, SY006 and SY009 were shown to be significantly more toxic than SY007. Cytotoxic effects of PWO381, PWO577 and SY007 were detected, but only at very high concentrations, way above those normally used in activity assays^19^. In summary, the presented in vitro cytotoxicity experiments allowed for the selection of compounds with good cytotoxicity profiles from both EPAC1 ligand series. In the *N*-acylsulfonamide class, PWO381 and PWO577 were found to be best tolerated by HUVECs, whereas, among the benzofuran oxoacetic acid derivatives (SY series), compound SY007 was significantly less toxic than SY006 and SY009. In combination with their binding properties and demonstrated in-cell activities, this makes PWO577 and SY007 promising candidates for further drug development and in-depth activity studies in cellulae.

From RNAseq and proteomic experiments we found that PWO577 and SY007 exerted qualitatively similar effects on gene expression, leading to upregulation of a subset of MMP1 at the mRNA and protein level (Figs. 4 and 5). Beyond this, many of the induced gene expression changes detected by RNAseq led to only modest changes in protein expression (e.g., ICAM1) or were absent from proteomic analysis (e.g., CCL2). Given that PWO577 and SY007 treatment led to many more changes in protein expression (Fig. 5), beyond those identified by RNseq (Fig. 4), implies that EPAC1 activation may also promote post-transcriptional mechanisms of gene regulation, in addition to the transcriptional changes identified here. Another point to consider, is that RNAseq and proteomic experiments were carried out using the same time points. This may explain why the results don’t map exactly to each other, since changes in RNA levels normally precede changes in protein translation. This will need to be investigated in future.

EPAC1 has emerged as an important factor in the regulation of the pro-inflammatory IL-6 *trans*-signalling pathway in VECs^35–38^, including promotion of SOCS3 induction and blockade of IL-6 JAK/STAT3 signalling, suppression of the expression of the pro-inflammatory cell adhesion molecule, VCAM1^39^. Moreover, EPAC1 protects the heart from cytokine-induced cardiac dysfunction, at least in part, through the inhibition of the JAK-STAT pathway^40^, and decreases foam cell formation^41^, although contradictory data to this point has recently been published^42^. Here we show that PWO577 and SY007 suppresses STAT3 activation by IL-6 and downstream gene expression, including SOCS3, IL6ST, STAT3 and JAK3 (Fig. 7). This probably arises from early upregulation of SOCS3 and inhibition of JAK signalling to STAT3 as demonstrated for IL-6 signalling in HUVECs^22^. Notably, PWO577 and SY007 were not able to inhibit CCL2 and ICAM1 induction by IL-6 (Fig. 7). This is probably because inhibition of STAT3 by PWO577 and SY007 is insufficient to block induction of these two genes. Indeed, for ICAM1 we have previously shown that inhibition of ERK and PI3K induces expression of ICAM1 in I942-stimulated HUVECs^22^. A similar situation might underly the regulation of CCL2, but this remains to be determined, moreover upregulation of CCL2 mRNA may necessarily lead directly to up-regulation of CCL2 protein, as shown in Figs. 4 and 5. It is tempting to speculate that wider gene expression changes induced by PWO577 and SY007 here might involve activation of the AP1/Jun transcription factor complex as described for I942^43,44^. This is a particularly attractive idea of one study suggesting that AP1/Jun transcription factors play a key role in the control of transcriptional networks in human aortic endothelial cells by co-binding enhancers with VEC-specific genes^45^. Whether these mechanisms are further controlled by EPAC1 activation remains to be determined.

In summary, we have identified PWO577 and SY007 as EPAC1 activators that are specific, non-toxic and inhibit IL-6 activation in HUVECs. Further modifications to the compounds will be guided by these results to eliminate the few noted off target effects identified.

## Methods

### Materials

8-(4-chlorophenylthio)-2′-*O*-methyladenosine-3′,5′-cyclic monophosphate (D-007) was purchased from Biolog Life Science Institute, Bremen, Germany. 5-carboxyfluorescein diacetate acetoxymethyl ester (CFDA-AM), Alamar blue cell viability reagent and SYPRO™ Orange protein stain were purchased from ThermoFisher Scientific, Waltham, MA, USA. Adenosine 3′,5′-cyclic monophosphate (cyclic AMP) sodium salt, 0.33% neutral red solution, dimethyl sulfoxide (DMSO) for molecular biology and anti-Rabbit IgG, HRP conjugate, were obtained from Sigma-Aldrich, St. Louis, MO, USA. Rap1A/Rap1B (26B4) rabbit mAb, EPAC1 (DE3) mouse mAb and STAT3/phospho-STAT3 (Tyr 705) antibodies were purchased from Cell Signaling Technology, Danvers, MA, USA.

### Chemical synthesis

Compounds I942, PW0381, PW0521 and PWO577 were synthesised as previously described (compounds 3, 25g, 25q, 25n, respectively, in^21^). Compounds SY006, SY007 and SY009 were synthesised as previously described^19^.

### Cell culture

Cryopreserved primary human umbilical vein endothelial cells (HUVECs) as well as dedicated medium and growth supplements, were bought from (PromoCell, Heidelberg, Germany). Cells were cultured in the growth medium, comprising Endothelial Cell Growth Basal Medium supplemented with Endothelial Cell Growth Medium SingleQuots Supplements (excluding antibiotics), in 75 cm^2^, tissue culture treated flasks, at 37 °C, in a humidified atmosphere containing 5% (v/v) CO_2_. HUVECs were used for experiments at no older than passage 6.

Human bone osteosarcoma epithelial cells (U2OS) stably transfected to express FLAG-tagged EPAC1 were a gift from Professor Holger Rehmann (Flensburg University of Applied Sciences, Germany). Cells were cultured in 75 cm^2^, tissue culture treated flasks containing complete medium, comprising Dulbecco's modified Eagle's medium (DMEM), with 4.5 g/l d-glucose and phenol red, supplemented with 10% (v/v) foetal bovine serum (FBS), 1% (v/v) GlutaMAX and 1% (v/v) Penicillin–Streptomycin (all from ThermoFisher Scientific), at 37 °C in a humidified atmosphere containing 5% (v/v) CO_2_. To ensure selection of stable transfectants, 2 mg/l puromycin was also added to the complete medium.

### Protein purification

A pGEX-6P-1 vector, expressing GST-EPAC1-CNBD (amino acids 169–318)^20^ and pGEX-5X-1 expressing GST-RalGDS-RBD (788–884)^44^, were transformed into *E. coli* One Shot BL21 Star (DE3) cells (ThermoFisher Scientific), following the manufacturer’s protocol. Protein expression was induced by addition of IPTG and followed by the affinity purification of the EPAC1-CNBD (169–318) or RalGDS-RBD (788–884) GST fusion proteins as previously described^20^. Protein concentration was measured using a NanoDrop 2000/2000c (ThermoFisher Scientific).

### Thermal shift assays

Recombinant GST-EPAC1-CNBD (2 μM), test ligand dilutions (3.1–100 μM) or 0.0006% to 0.02% (v/v) DMSO dilutions (diluent for ligands), were combined with 1 × (v/v) SYPRO Orange dye in PCR-compatible microplates, which were then stored, protected from light, overnight at 4 °C. Plates were then subjected to a standard temperature gradient programme using a real-time PCR machine (Applied Biosystems StepOnePlus Real-time PCR Instrument, ThermoFisher Scientific). The applied temperature range was 11–80 °C, ramping up by 0.5 °C increments, with a 30-s hold at each temperature. Changes in the fluorescence of SYPRO Orange dye were detected using a filter set for a ROX reporter dye (ex/em maxima 580/621 nm). Melting temperatures (Tm) were generated from melt curves by fitting a selected range of acquired data to a Boltzmann equation, using the first derivative approach^26^.

### Cytotoxicity assays

The method used here is a modified version of the protocol developed by Dayeh et al.^46^, which allows the simultaneous use of three different fluorescence-based cytotoxicity assays, using the same set of cells: Alamar Blue assay, 5-carboxyfluorescein diacetate acetoxymethyl ester (CFDA-AM) assay, and Neutral Red assay. This allows the simultaneous determination of the impact of each compound on cell metabolic activity, plasma membrane integrity, and lysosomal function. The half-maximal effective concentration (EC_50_) parameter was adopted as a measure of cytotoxic potential of test compounds in each assay. To obtain EC_50_ values from the dose–response data, curve fitting was performed using nonlinear regression in GraphPad Prism 8 software.

### EPAC1 and Rap1 activation assays

U2OS cells stably transfected with EPAC1 were stimulated with ligands and then washed with ice-cold 1 × PBS and then lysed in 0.5 ml of Rap1 Assay Lysis Buffer (55 mM Tris–HCl, pH 7.4,132 mM NaCl, 22 mM NaF, 11 mM, Na_4_P_2_O_7_, 10 mM MgCl_2_, 1% (v/v) Triton X-100) supplemented with 1 mM phenylmethylsulfonyl fluoride (PMSF), followed by centrifugation at 16,000×*g* for 15 min at 4 °C to clear the cell lysates and then incubated with 40 µg of GST-RalGDS-RBD immobilized on Glutathione Sepharose 4B (GE Healthcare, Chicago, IL, USA), for 1 h at 4 °C on a rotator, to selectively capture active Rap1. After incubation, the glutathione resin was separated from the supernatant by centrifugation at 500×*g* for 5 min at 4 °C and then the beads were washed 3 times with 0.4 ml of Rap1 Assay Lysis Buffer. The beads were then resuspended in 2 × SDS sample loading buffer and denatured by heating for 5 min at 95 °C. Prepared pull-down and input control samples were then subjected to SDS-PAGE and western blotting with an anti-Rap1 antibody to detect Rap1.GTP levels. To immunoprecipitate active EPAC1, 2 µl of the EPAC1 (5D3) mouse mAb was added to cleared lysates and incubated for 30 min at 4 °C on a rotator. Following this, 10 µl of protein G magnetic beads (New England Biolabs, Ipswich, USA) was added to each lysate containing the antibody, followed by a further 1-h incubation on a rotator at 4 °C. Protein G magnetic beads were captured from the supernatant using a magnetic separation rack and then washed 3 times with 0.5 ml of ice-cold RIPA buffer. Prepared IP and input control samples were then subjected to SDS-PAGE and western blotting, to detect levels of active EPAC1.

### SDS-PAGE and western blotting

Samples for SDS-PAGE were prepared by scraping the cells directly in 1 × SDS Sample Loading Buffer (125 mM Tris–HCl, pH 6.8, 4% (w/v) SDS, 20% (v/v) glycerol, 0.02% (w/v) bromophenol blue, 20 mM DTT) and then denaturing for 5 min at 95 °C. Protein samples and a broad-range pre-stained protein marker (11–190 kDa; BioRad) were loaded onto polyacrylamide gels and electrophoresis was carried out in 1 × Running Buffer (25 mM Tris–HCl, 192 mM glycine, 0.1% (w/v) SDS) at 80 V for the first 30 min and then at 130 V for the next 50–70 min. After separation by SDS-PAGE proteins were transferred from gels to nitrocellulose membranes in 1 × Transfer Buffer (25 mM Tris–HCl, 192 mM glycine, 20% (v/v) methanol) at 80 V for 75 min. Following the transfer, membranes were washed in 1 × TBS (20 mM Tris–HCl pH 7.4, 150 mM NaCl) for 5 min and then blocked for 1 h at room temperature in 5% (w/v) non-fat dry milk in 1 × TBS-T (20 mM Tris–HCl pH 7.4, 150 mM NaCl, 0.1% (v/v) Tween-20). The blocking step was followed by overnight incubation with primary antibody diluted 1:1000 in the blocking buffer at 4 °C, followed by incubation with horseradish peroxidase-conjugated anti-rabbit secondary antibody diluted in 5% (w/v) non-fat dry milk in 1 × TBS-T for 1 h at room temperature. Membranes were then washed three times with 15 ml of 1 × TBS-T and then incubated for 5 min with SuperSignal West Pico PLUS Chemiluminescent Substrate (ThermoFisher Scientific). Images were acquired using a Fusion FX7 camera platform (Vilber, Collégien, France) on the chemiluminescence setting. Signal intensities were measured densitometrically, using ImageJ software (National Institutes of Health, Bethesda, USA), and normalised to the signal obtained from a known housekeeping protein in the same sample.

### RNA sequencing

HUVEC cells were grown on 6-well plates until they had achieved 70–80% confluence. Cells were then pre-incubated for 30 min in the presence or absence of 100 μM PWO577 or SY007 for 30 min, following treatment with IL6 (5 ng/ml) plus IL6Rα (25 ng/ml) for 16 h. Total RNA was isolated from cells using RNeasy Mini Kit (Qiagen, Manchester, UK), according to the manufacturer's protocol. RNA concentration was determined using NanoDrop 1000 Spectrophotometer (Thermo Fisher Scientific, Paisley, UK). RNA sample quality control, RNA library preparation, library quality control, Illumina library sequencing, data quality control and bioinformatics analysis were then carried out by Novogene UK (Cambridge, UK). In brief, raw reads of FASTQ format were firstly processed through in-house Perl scripts. In this step, clean data (clean reads) were obtained by removing reads containing adapter, reads containing poly-N, and low-quality reads from raw data^47^. All the downstream analyses were based on the clean data with high quality. Paired-end clean reads were aligned to the human genome using Hisat2 v2.0.5^48^. Then, the abundance of each transcript was quantified using FeatureCounts v1.5.0-p3^49^. Differentially expressed gene (DEGs) analysis was performed using the DESeq2 package^50^ and gene expression was normalized using relative-log-expression (RLE). Genes with an adjusted p-value < 0.05 were assigned as being differentially expressed. Metascape (http://metascape.org) was employed to perform gene enrichment and functional annotation analyses by sourcing the Kyoto Encyclopaedia of Genes and Genomes (KEGG) Pathway^51,52^. The profileR package^53^ was used to test the statistical enrichment of differential expression genes in KEGG pathways.

### Proteomic analysis

HUVEC cells were grown on 10 cm dishes until they had achieved 70–80% confluence. Then, the cells were treated with 100 µM of PWO577 or SY007 for 16 h. Cells were then washed with PBS, scrapped into 1 ml of PBS and centrifuged 5 min at 800 rpm. Cell pellets were collected and frozen at − 80 °C. Proteomic analysis was then carried out by DC Biosciences Ltd (Dundee, UK). Briefly, peptides were labelled with TMT 10-plex reagents according to the instructions provided by the manufacturer (Pierce, Rockford Eppendorf tubes). Tryptic peptides were subjected to LC-MSMS analysis on an Orbitrap Fusion Lumos mass spectrometer (ThermoFisher Scientific, San Jose, CA) coupled to a Dionex Ultimate 3000RSLC nano system (ThermoFisher Scientific, San Jose, CA) via a nanoflex ion source. Analysis was performed in a data-dependent acquisition mode with MaxQuant v. 2.1.0.0. Raw files were searched against the latest human SwissProt FASTA database containing only canonical isoforms (version 05.2022; 20,376 sequences) downloaded from UniProt.

### Statistical analysis

One-way analysis of variance (ANOVA) with Tukey’s or Dunnett’s post hoc test were performed using GraphPad Prism 9 software (GraphPad Software, San Diego, USA).

## Supplementary Information

Supplementary Information.

## Data Availability

All data generated or analysed during this study are included in this published article and its supplementary information file.
